# Broad Anti-Viral Capacities of Lian-Hua-Qing-Wen Capsule and Jin-Hua-Qing-Gan Granule and Rational use Against COVID-19 Based on Literature Mining

**DOI:** 10.3389/fphar.2021.640782

**Published:** 2021-05-14

**Authors:** Mingfei Shi, Bo Peng, An Li, Ziyun Li, Ping Song, Jing Li, Ruodan Xu, Ning Li

**Affiliations:** ^1^ Institute of Basic Theory for Chinese Medicine, China Academy of Chinese Medical Sciences, Beijing, China; ^2^ State Key Laboratory of Quality Research in Chinese Medicine, Institute of Chinese Medical Sciences, University of Macau, Macao, China; ^3^ The Third School of Clinical Medicine, Nanjing University of Chinese Medicine, Nanjing, China; ^4^ Guang'anmen Hospital, China Academy of Chinese Medical Sciences, Beijing, China; ^5^ Department of Nephropathy, Dongzhimen Hospital, Beijing University of Chinese Medicine, Beijing, China

**Keywords:** broad-spectrum antivirals, Lian-Hua-Qing-Wen capsule, Jin-Hua-Qing-Gan granule, medicinal plants, COVID-19, SARS-CoV-2, host-directed therapy

## Abstract

The novel coronavirus disease 2019 (COVID-19) has become a matter of international concern as the disease is spreading exponentially. Statistics showed that infected patients in China who received combined treatment of Traditional Chinese Medicine and modern medicine exhibited lower fatality rate and relatively better clinical outcomes. Both Lian-Hua-Qing-Wen Capsule (LHQWC) and Jin-Hua-Qing-Gan Granule (JHQGG) have been recommended by China Food and Drug Administration for the treatment of COVID-19 and have played a vital role in the prevention of a variety of viral infections. Here, we desired to analyze the broad-spectrum anti-viral capacities of LHQWC and JHQGG, and to compare their pharmacological functions for rational clinical applications. Based on literature mining, we found that both LHQWC and JHQGG were endowed with multiple antiviral activities by both targeting viral life cycle and regulating host immune responses and inflammation. In addition, from literature analyzed, JHQGG is more potent in modulating viral life cycle, whereas LHQWC exhibits better efficacies in regulating host anti-viral responses. When translating into clinical applications, oral administration of LHQWC could be more beneficial for patients with insufficient immune functions or for patients with alleviated symptoms after treatment with JHQGG.

## Introduction

### Lian-Hua-Qing-Wen Capsule and Jin-Hua-Qing-Gan Granule are Both Recommended as Effective “Chinese Solution” Against COVID-19

The novel coronavirus disease 2019 (COVID-19) pandemics has reached almost every country in the world. Compared with the outbreak of Severe Acute Respiratory Syndrome (SARS) in 2003 and the pandemic of Middle East Respiratory Syndrome (MERS) in 2012, COVID-19 caused by the novel coronavirus SARS-CoV-2 infection has relatively low fatality rate, whereas much more rapid and higher human-to-human transmissibility (Meo et al., 2020). Typically, the existence of a large number of asymptomatic carriers of SARS-CoV-2 additionally exerts potential burden to the control and prevention of COVID-19.

SARS-CoV-2 can be easily transmitted through respiratory droplets or by aerosol, and infected people have a wide range of reported symptoms, from mild symptoms to severe illness. The most common manifestations of COVID-19 are fever or chill, dry cough and fatigue, which could be accompanied with a temporary loss of smell or taste, muscle or body aches. In critical cases, acute myocardial injury, liver or kidney dysfunction and blood-clotting complications may occur Huang et al. (2020), Khider et al. (2020), consequently leading to septic shock and acute respiratory distress syndrome (ARDS) or death. The “Clinical Treatment for COVID-19” issued by the World Health Organization recommends that symptomatic treatments that relieve fever and pain, together with adequate nutritional supports are basically required for mild cases of COVID-19. For severe SARS-CoV-2 infections, oxygen therapy and fluid supply need to be reinforced. In spite of supportive measures above, potential anti-viral drugs which were used for diseases due to viral infections other than SARS-CoV-2 have been repurposed for COVID-19, such as remdesivir, ribavirin and hydroxychloroquine are however not addressed because of reported side-effects or lack of supporting evidence from large-scale randomized controlled trials (Izcovich et al., 2020; Trivedi et al., 2020; Qaseem et al., 2021). Likewise, vaccine development involves a difficult, complex and costly process, and the success of which is at a high risk of failure protecting against mutant viral variants (Biswas and Majumder, 2020; Penarrubia et al., 2020). Despite the development of vaccines, scientists are still tirelessly designing new drugs and repurposing existing drugs against SARS-CoV-2. Though tremendous strides have been made in the fight against coronaviruses, a lack of safe and effective anti-SARS-CoV-2 drugs is still a key factor restricting the prevention and control of COVID-19 pandemics.

The practice of Traditional Chinese Medicine (TCM) has accumulated a wealth of clinical experience in the treatment of infectious diseases since Qin-Han (about 221 BC to 220 AD) and developed into a theory in Ming-Qing period (about 1,368–1777 AD). Infectious diseases in TCM have been described as “infections caused by toxic qi”, “warm pathogen first invades lung via nose and mouth”, and “disease spreads due to close contact”. These descriptions fit well with the epidemiological characteristics of modern acute infectious diseases. According to TCM theory, COVID-19 is the result of invasion by dampness-toxin pathogens, therefore COVID-19 is pathogenically characterized by dampness-toxin and host healthy-qi deficiency. Most patients first present mild sign of dampness, like fatigue, poor appetite and greasy thick tongue coating (Zheng, 2020). As disease progresses, dampness-toxin invades interiority and diffuses into triple energizer, leading to vital qi impairment and accumulation of toxin-qi in viscera. Excessive accumulation of dampness-toxin may easily lead to vital qi exhaustion and consequently loss of life. Hence, TCM formulae functioning to remove dampness-toxin are effective in preventing COVID-19 progress. Being the first country that was attacked by COVID-19, approximately 91.5% confirmed patients in China were treated with TCM formulae and the total effective rate has reached to 90%. In Wuhan Jiang-Xia Square Cabin Hospital, none of the 564 COVID-19 patients who received combined treatment of TCM and modern medicine developed into severe conditions, and TCM addition significantly reduced the course of hospitalization (Ren et al., 2020).

Both LHQWC and JHQGG belong to “Three Drugs, Three Prescriptions”, official prescriptions of TCM used in the fight against COVID-19 in China. LHQWC, composed of *Forsythia suspensa* (Thunb.) Vahl, *Lonicera japonica* Thunb*.*, honey-fried *Ephedra sinica* Stapf, fried *Prunus sibirica* L*.*, Gypsum Fibrosum*, Isatis tinctoria* L*., Dryopteris crassirhizoma* Nakai*, Houttuynia cordata* Thunb*., Pogostemon cablin* (Blanco) Benth*., Rheum palmatum* L*., Rhodiola crenulata* (Hook.f. and Thomson) H. Ohba*, Mentha canadensis* L*.* and *Glycyrrhiza glabra* L*.*, is innovative Chinese Patent Medicine (CPM) approved during the SARS epidemics in 2003. JHQGG, the other CPM constituting *Forsythia suspensa* (Thunb.) Vahl*, Lonicera japonica* Thunb*., Ephedra sinica* Stapf*, Prunus sibirica* L*.,* l-Menthol*, Glycyrrhiza glabra* L*., Scutellaria baicalensis* Georgi*, Fritillaria thunbergii* Miq*., Anemarrhena asphodeloides* Bunge*, Arctium lappa* L*.* and *Artemisia annua* L*.,* has been approved to treat H1N1 influenza virus infection since 2009. Both LHQWC and JHQGG are developed based on Ma-Xing-Shi-Gan Decoction and Yin-Qiao Powder, classic TCM decoctions used for respiratory infections recorded in *Treatize on Exogenous Febrile Disease* (about 210 AD) and *Systematic Differentiation of Warm Diseases* (1798 AD), respectively. In clinical practices resolving respiratory infections, LHQWC is mainly used to clear away plague, remove toxins, ventilate lungs and discharge heat, whereas JHQGG is applied to dispel wind, clear heat and resolve toxin. In the combat against COVID-19, National Health Commission of China approved both LHQWC and JHQGG as clinical therapies in China, and observational studies showed that both can effectively relieve fever, fatigue, cough and phlegm in the early stage of COVID-19, contributing to reductions in risks of rapid clinical deterioration. Supportively, *in vitro* studies have revealed that both formulae have anti-inflammatory effects, providing fundamental evidence for clinical application of both formulae in the fight against COVID-19 (Cheng, 2020; Duan, 2020; Hu et al., 2020; Runfeng et al., 2020; Zhang et al., 2020).

### Holism Theory of TCM and Anti-viral Actions of Lian-Hua-Qing-Wen Capsule and Jin-Hua-Qing-Gan Granule, a Reflection of Host-Directed Therapy in Modern Medicine

Holism is the fundamental concept in TCM, which emphasizes the connections of the whole body and intends to treat the whole person rather than focusing on individual symptoms. Directed by holistic view, TCM practitioners adopt syndrome differentiation (Bian Zheng), a comprehensive analysis of a variety of clinical information, and herbal formulae to resolve single or complex uncomfortability of patients. This holism theory of TCM dovetails with the principle of host-directed therapy (HDT). HDT is a novel concept in the treatment for infectious diseases and was first used in *tuberculosis* in 2015 (Zumla et al., 2015). After then, HDT was gradually fulfilled as anti-viral strategies. Compared to conventional anti-viral therapies, which focus on inhibiting virus activity, HDT aims to maintain homeostasis of host by stimulating anti-viral responses and suppressing immune injuries. It has been shown that compared to single anti-pathogen treatment, HDT is able to reduce the risks of drug resistance induced by bacteria and viruses, endowing HDT a therapeutic potential of being broad-spectrum anti-viral tactics (Kaufmann et al., 2018). Clinical investigations proposed that viral infection-triggered cytokine storm was a vital factor mediating the rapid progress of COVID-19 (Wang T. et al., 2020). High levels of IL (Interleukin) -6 and IL-10, while low levels of CD4^+^ T and CD8^+^ T cells can be observed in COVID-19 patients (Guan et al., 2020; Wan et al., 2020). Moreover, plasma IL-2, IL-7, IL-10, GCSF (granulocyte colony-stimulating factor), IP-10 (interferon gamma-induced protein-10), MCP-1 (monocyte chemoattractant protein-1), MIP-1α (macrophage inflammatory protein-1 alpha) and TNF-α (tumor necrosis factor-alpha) are consistently higher in intensive care unit (ICU) patients compared to mild cases (Huang et al., 2020), suggesting that virus-induced exaggerated immune responses and the resulting immune injuries are involved in the progression of COVID-19. Accordingly, HDT-oriented treatments that inhibit IL-6 signaling by down-regulating IL-6 receptors have been suggested as a potential solution for COVID-19 patients (Zumla et al., 2020). Consistent with HDT, in the combat against COVID-19, TCM addresses that sufficient healthy-qi within the body is key to prevent pathogen invasion, so-called “strengthening host resistance to eliminate pathogenic factors”. Accordingly, inspiring vital qi is at the root of preventing infectious diseases in TCM. The functions of “healthy-qi” resemble “immunity” of host, and “pathogenic factors” stand for all substances that affect host homeostasis, such as viruses and bacteria. As emphasized in HDT that considering individuals as a whole rather than separating parts, “strengthening host resistance to eliminate pathogenic factors” in TCM addresses an overall reaction of host in response to invasive viruses, whereas the destiny of pathogen itself is not primarily important. Moreover, same as the HDT concept implicates, the ultimate goal of TCM treatment is to maintain host homeostasis *via* balancing interactions between host and pathogens, or by establishing equilibrium between stimulating anti-viral reactions and suppressing overactivated immune responses that subsequently cause tissue injuries.

Following the HDT principle and holism theory of TCM, this study primarily desired to gain more insight into the broad anti-viral features of LHQWC and JHQGG, both of which have been applied to treat a variety of viral infections. However, considering that the main herbal composition of LHQWC and JHQGG largely overlap, it therefore appears confusing in the selection of appropriate formula for individual clinical cases. In this scenario, it is of prime importance to also distinguish the similarities and differences between the two formulae in terms of pharmacological anti-viral functions. To implement these goals, we manually grouped the individual active components from either LHQWC or JHQGG or both into two categories, namely constituents that interfere with viral life cycle and components that regulate host immune responses and inflammation. Through comprehensive literature review, data mining and pharmacological target enrichment analysis, we investigated the strength of LHQWC and JHQGG in the above-mentioned virus or host arm to compare their anti-viral functionalities. The holism-directed analysis of LHQWC and JHQGG will provide more insightful information and comprehensive understanding for rational use of these two CPMs in the combat against COVID-19, as well as the emerging or re-emerging pandemics of infectious diseases.

## Materials and Methods

### Literature Collection and Inclusion

In order to collect sufficient data on anti-viral effects of LHQWC and JHQGG, we employed Pubmed (https://pubmed.ncbi.nlm.nih.gov), Ovid (https://ovidsp.ovid.com/), CNKI (https://www.cnki.net), WANFANG (http://www.wanfangdata.com.cn/index.html) and WEIPU (http://www.cqvip.com/) database by searching either the full name of formulae, such as “Lianhua Qingwen Capsules”, “Jinhua Qinggan Granules”, or names of individual medicinal herbs, or active ingredients, together with “virus” as keywords. In addition, bioactive components that were proposed to be antivirals were included *via* network pharmacology-based prediction and analysis. A total of 1,110 articles were collected for next filtration. For the analysis of broad anti-viral activities, we then excluded studies reporting negative outcomes, clinical trials generally indicating viral infections without clarifying taxonomy of viruses, investigations using inactivated or attenuated viruses as vaccines, and articles with no access to full context due to age. A total of 812 articles were analyzed at this stage. For detailed comparisons of active anti-viral components and pharmacological functions of formulae, studies without indicating names of active components were further excluded. Notably, no information regarding Gypsum Fibrosum and fried *Prunus sibirica* L*.* in relevant to virus, and we did not find data by searching bioactive components directly isolated from JHQGG, hence we only took ingredients determined by predictive parsing of network pharmacology. Finally, 117 articles were included for comparison of pharmacological functions.

### Constructing “Formula–Herb–Virus–Baltimore Classification of Viruses” Network

In order to describe broad-spectrum anti-viral activities of LHQWC and JHQGG, we grouped antiviral data collected as mentioned, and built a network in forms of “Formula-herb-virus-Baltimore classification of viruses”. To further interpret the common and distinctive anti-viral activities of LHQWC and JHQGG in terms of holism theory of TCM, we classified the anti-viral actions reported for LHQWC and JHQGG into being either associated with viral life cycle or responsible to host immune responses and inflammation. To gain more insightful understanding, we further categorized active components that disrupt virus life cycle into three levels, including direct virucidal activity, inhibition of viral entry, and suppression of viral replication and egress. Generally, inhibitors of virus entry act through deforming viral particles or blocking the attachment or binding of virions to host cells. The control of virus replication is mainly mediated by inhibiting replicator machineries encoded by viral systems, and prevention of virus egress is a process involves an interference with assembly and release of progeny viruses, which may initiate a secondary round infection. For the actions of regulating host immune responses and inflammation, it represents any virucidal effects due to an indirect response by modulating host immune system, such as increasing interferons (IFNs) expression, or decreasing self-targeted inflammatory injuries, or promoting repair process post virus infection without involving viral molecule-associated biological events. Based on literature mining and analysis, we next counted the frequencies of active components of LHQWC and JHQGG that have been sorted into each of the two categories, and accordingly a radar chart was drawn to visualize and compare the power of LHQWC and JHQGG against viral infection in terms of modulating viral life cycle and regulating host immune responses and inflammation.

## Results

### The broad-Spectrum Anti-Viral Activities of Lian-Hua-Qing-Wen Capsule and Jin-Hua-Qing-Gan Granule

**FIGURE 1:**
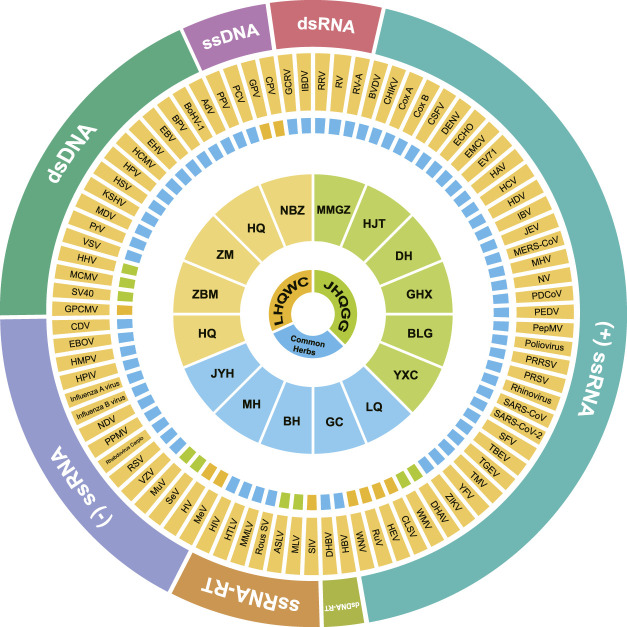
The broad-spectrum anti-viral activities of LHQWC and JHQGG. The “Formula–herb–virus–Baltimore classification of viruses” profile demonstrating a broad-spectrum anti-viral activity of LHQWC and JHQGG. In the center, medicinal herbals exclusively existing in LHQWC, including HQ (*Scutellaria baicalensis* Georgi, Huang Qin); ZBM (*Fritillaria thunbergii* Miq., Zhe Bei Mu); ZM (*Anemarrhena asphodeloides* Bunge, Zhi Mu); QH (*Artemisia annua* L., Qing Hao) and NBZ (*Arctium lappa* L., Niu Bang Zi) are shown in orange; medicinal herbals found only in JHQGG, including MMGZ (*Dryopteris crassirhizoma* Nakai, Mian Ma Guan Zhong); HJT (*Rhodiola crenulata* (Hook.f. and Thomson) H. Ohba, Hong Jing Tian); DH (*Rheum palmatum* L., Da Huang); GHX (*Pogostemon cablin* (Blanco) Benth., Guang Huo Xiang); BLG (*Isatis tinctoria* L., Ban Lan Gen) and YXC (*Houttuynia cordata* Thunb., Yu Xing Cao); are presented in green; common herbs used in both LHQWC and JHQGG, including LQ (*Forsythia suspensa* (Thunb.) Vahl*,* Lian Qiao); GC (*Glycyrrhiza glabra* L*.,* Gan Cao); *BH* (*Mentha canadensis* L*.,* Bo He); MH (*Ephedra sinica* Stapf*,* Ma Huang) and JYH (*Lonicera japonica* Thunb., Jin Yin Hua) are colored in blue. The circle marked in orange represents 87 types of viruses, and the cycle in the periphery indicates Baltimore classification of these viruses. Colored squares sitting between the circle of individual herbs and 87 viruses indicate that components existing only in LHQWC (orange) or only in JHQGG (green) or in both formulae (blue) have been reported effective to treat diseases caused the corresponding viruses. AdV, Adenoviruses; ASLV, Avian sarcoma leukosis virus; BoHV, Bovine alphaherpesvirus; BPV, Bovine papillomavirus; BVDV, Bovine viral diarrhea virus; CDV, Canine distemper virus; CHIKV, Chikungunya virus; CLSV, Cucumber leaf spot virus; Cox A, Coxsackie A virus; Cox B, Coxsackie B virus; CPV, Canine parvovirus; CSFV, Classical swine fever virus; DENV, Dengue virus; DHAV, Duck hepatitis A virus; DHBV, Duck hepatitis B virus; EBOV, Ebola virus; EBV, Epstein–Barr virus; ECHO, Echovirus; EHV, Equine herpes virus; EMCV, Encephalomyocarditis virus; EV71, Enterovirus A 71; GCRV, Grass carp reovirus; GPCMV, Guinea pig cytomegalovirus; GPV, Goose parvovirus; HAV, Hepatitis A virus; HBV, Hepatitis B virus; HCMV, Human cytomegalovirus; HCV, Hepatitis C virus; HDV, Hepatitis D virus; HEV, Hepatitis E virus; HHV, Human herpesvirus; HIV, Human immunodeficiency virus; HMPV, Human metapneumovirus; HPIV, Human parainfluenza virus; HPV, Human papillomavirus; HSV, Herpes simplex virus; HTLV, Human T lymphotropic virus; HV, Hantavirus; IBDV, Infectious bursal disease virus; IBV, Infectious bronchitis virus; JEV, Japanese encephalitis virus; KSHV, Kaposi's sarcoma herpesvirus; MCMV, Murine cytomegalovirus; MDV, Marek's disease virus; MERS-CoV, Middle East respiratory syndrome coronavirus; MHV, Mouse Hepatitis virus; MLV, Murine leukemia virus; MMLV, Moloney Murine Leukemia virus; MuV, Mumps virus; NDV, Newcastle disease virus; NV, Norovirus; PCV, Porcine circovirus; PDCoV, Porcine deltacoronavirus; PEDV, Porcine epidemic diarrhea virus; PepMV, Potato–Pepino mosaic virus; PPV, Porcine parvovirus; PPMV, pigeon paramyxovirus; PPV, Pigeonpox virus; PRRSV, Porcine reproductive and respiratory syndrome virus; PRSV, Papaya ringspot virus; PrV, Pseudorabies virus; Rous SV, Rous sarcoma virus; RRV, Ross River virus; RSV, Respiratory syncytial virus; RuV, Rubella virus; RV, Rotavirus; RV-A, SA-11 Simian rotavirus; SARS-CoV, Severe acute respiratory syndrome coronavirus; SARS-CoV-2, Severe acute respiratory syndrome coronavirus 2; SeV, Sendai virus; SFV, Semliki Forest virus; SIV, Simian immunodeficiency virus; SV40, Simian virus 40; TBEV, Tick-borne encephalitis virus; TGEV, Transmissible Gastroenteritis virus; TMV, Tobacco mosaic virus; VSV, Vesicular stomatitis virus; VZV, Varicella zoster virus; WMV, Watermelon mosaic virus; WNV, West Nile virus; YFV, Yellow fever virus; ZIKV, Zika virus. RNA, Ribonucleic Acid; -ssRNA, Negative-sense single-strand RNA; +ssRNA, Positive-sense single-stranded RNA; dsRNA, Double-stranded RNA; ssRNA-RT, Single-stranded RNA virus-reverse transcriptase; DNA, Deoxyribonucleic Acid; ssDNA, Single-stranded DNA; dsDNA, Double-stranded DNA; dsDNA-RT, Double-stranded DNA virus-reverse transcriptase.

Multi-ingredients, multi-targets and multi-pathways are primary features of TCM formulae, suggesting that active ingredients of one medicinal herb may exert anti-viral functions *via* diverse pharmacological mechanisms. As shown in Figure 1, active components in both LHQWC and JHQGG have been shown to target 87 different types of viruses, covering all the seven classes according to the Baltimore classification. This wide range of anti-viral activities of LHQWC and JHQGG addresses that TCM formulae used in COVID-19 pandemics could be potentially applied for other virological infections, such as influenza A virus, Zika virus and herpesvirus.

### Similarities and Differences of Lian-Hua-Qing-Wen Capsule and Jin-Hua-Qing-Gan Granule as Antivirals

**FIGURE 2:**
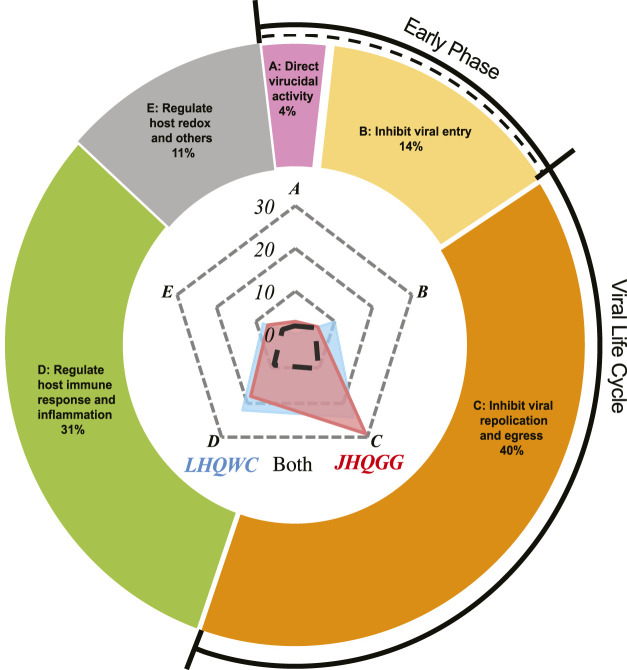
Comparison of anti-viral mechanisms between LHQWC and JHQGG. Anti-viral potentials of LHQWC and JHQGG are grouped into five categories, which are defined as **(A)**. Direct virucidal activity, **(B)**. Inhibit viral entry, **(C)**. Inhibit viral replication and egress, **(D)**. Regulate host immune responses and inflammation and **(E)**. Regulate host redox and others”. The percentage in each category indicates the power of both LHQWC and JHQGG in individual anti-viral actions, among which the “A. Direct virucidal activity” and “B. Inhibit viral entry” belong to the early phase of viral infection as marked by black dotted line; the “A. Direct virucidal activity”, “B. Inhibit viral entry” and “C. Inhibit viral replication and egress”together constitute the whole viral life cycle, as surrounded in black. Comparation of LHQWC and JHQGG is demonstrated in the center, with actions from components only in LHQWC shown in blue, only of JHQGG in red, and for both LHQWC and JHQGG are circled within the black dotted area. 0–40 represents counted frequencies of either LHQWC or JHQGG in each of the five categories.

Both LHQWC and JHQGG possess broad-spectrum anti-viral potentials through interfering with viral life cycle and modulating host immune responses, which are associated with a diversity of proposed pharmacological actions as detailed in Tables 1, 2, 3; Figure 2. When comparing LHQWC and JHQGG, no difference was found in the types of their targeted viruses (Table 1; Figure 1). In terms of active components that disrupt viral life cycle (Table 1; Figure 2), only few literatures reported a direct virucidal activity from components of LHQWC and JHQGG (Table 1-1.1; Figure 2), about 24% studies showed suppression of viral entry (Table 1-1.2; Figure 2), while 70% studies focused on inhibitory effects toward viral replication and release (Table 1-1.3; Figure 2) Among all data analyzed, constituents from *Scutellaria baicalensis* Georgi (Huang Qin) of JHQGG have been mostly reported to interfere with viral life cycle in all three phases analyzed. Besides, components from *Isatis tinctoria* L (Ban Lan Gen) and *Rheum palmatum* L (Da Huang) of LHQWC are shown highly effective in blocking viral entry, replication and release. JHQGG weights slightly higher than LHQWC in terms of viral replication and release, whereas little difference was obtained in the early phase of viral life cycle (Table 1; Figure 2). Regarding “host immune responses and inflammation”, it is interesting that constituents from *Scutellaria baicalensis* Georgi (Huang Qin) of JHQGG again exhibited the greatest potential, followed by components from *Isatis tinctoria* L (Ban Lan Gen) and *Rheum palmatum* L (Da Huang) in LHQWC. When comparing LHQWC and JHQGG, LHQWC weights slightly higher than JHQGG (Table 2; Figure 2). In addition, several studies have proposed other anti-viral mechanisms that could not be grouped into the above two categories, such as maintaining host redox homeostasis, or acting on microbiota, or gut-lung axis, or energy sensor AMPK, or autophagy (Table 3; Figure 2). Detailed information regarding the TCM features, pharmacological functions of individual herbs and components was outlined in Table 4.

**TABLE 1:** Active anti-viral components from LHQWC and JHQGG, and their mechanisms of action regulating viral life cycle.

1.1 Direct virucidal activity			
Virus	Active component	Herb	References
Chikungunya Virus	Baicalin	*Scutellaria baicalensis* Georgi (Huang Qin)	Oo et al. (2018)
Coxsackievirus A16	Glycyrrhizic acid	*Glycyrrhiza glabra* L. (Gan Cao)	Wang et al. (2013)
Herpes simplex virus type1	Chinonin/Asphonin	*Anemarrhena asphodeloides* Bunge (Zhi Mu)	Jiang and Xiang (2004)
Newcastle disease virus	Baicalin	*Scutellaria baicalensis* Georgi (Huang Qin)	Jia et al. (2016)
Respiratory syncytial virus	*Lonicera japonica* Thunb extracts	*Lonicera japonica* Thunb. (Jin Yin Hua)	Zhang et al. (2014)

**1.2 Inhibit viral entry**				
Virus	Active component	Mechanisms	Herb	Ref
Coxsackie virus B3	Artemisinin	Inhibits viral absorption	*Artemisia annua* L. (Qing Hao)	Ma (2004)
	Baicalin	Reduces cellular lipid synthesis	*Scutellaria baicalensis* Georgi (Huang Qin)	Wang et al. (2020a)
Herpes simplex virus	*Houttuynia cordata* Thunb. Extracts	Blocks viral binding and penetration	*Houttuynia cordata* Thunb. (Yu Xing Cao)	Zhou (2017); Hung et al. (2015)
Herpes simplex virus type1	*Isatis tinctoria* L. extracts	Inhibits viral entry	*Isatis tinctoria* L. (Ban Lan Gen)	Fang, 2005)
Herpes simplex virus type1 type2 and varicella zoster virus	Houttuynoid A	Blocks viral membrane fusion	*Houttuynia cordata* Thunb. (Yu Xing Cao)	Li et al. (2017a)
Herpes simplex virus type2	Chinonin/Asphonin	Inhibits viral adsorption	*Anemarrhena asphodeloides* Bunge (Zhi Mu)	Jiang et al. (2005)
Human cytomegalovirus	Baicalein	Blocks viral entry through inhibiting epidermal growth factor receptor tyrosine kinase activity and viral nuclear translocation	*Scutellaria baicalensis* Georgi (Huang Qin)	Evers et al. (2005)
Human rotavirus	*Rheum palmatum* L. extracts	Inhibits viral entry	*Rheum palmatum* L. (Da Huang)	He et al. (2013)
Influenza A Virus	Flavonoids-enriched extract from *Scutellaria baicalensis root*	Reduces hemagglutinin	*Scutellaria baicalensis* Georgi (Huang Qin)	Zhi et al. (2019)
	Rhein	Inhibits viral absorption	*Rheum palmatum* L. (Da Huang)	Wang et al. (2018)
	*Isatis tinctoria* L. extract Clemastanin B, epigoitrin, phenylpropanoids portion and the mixture of *phenylpropanoids*, alkaloids and organic acid fractions	Blocks viral attachment	*Isatis tinctoria* L. (Ban Lan Gen)	Xiao et al. (2016)
	Glycyrrhizin	Reduces endocytotic activity and virus uptake	*Glycyrrhiza glabra* L*.* (Gan Cao)	Wolkerstorfer et al. (2009)
	*Isatis tinctoria* L. water extracts	Inhibits attachment of viruses to cells	*Isatis tinctoria* L. (Ban Lan Gen)	Chen et al. (2006)
	(+)-catechin	Inhibits acidification of endosomes and lysosomes	*Ephedra sinica* Stapf (Ma Huang)	Mantani et al. (2001)
	5,7,4′-trihydroxy-8-methoxyflavone	Inhibits fusion of virus with endosome/lysosome membrane	*Scutellaria baicalensis* Georgi (Huang Qin)	Nagai et al. (1995a); Nagai et al. (1995b)
Influenza A virus, Coxsackievirus B3, Adenovirus	Patchouli alcohol	Inhibits infection at the earliest stages of the viral life cycle, including virus attachment and entry	*Pogostemon cablin* (Blanco) Benth. (Guang Huo Xiang)	Wei et al. (2013)
Porcine reproductive and respiratory syndrome virus	Flavaspidic acid AB	Inhibits viral endocytosis	*Dryopteris crassirhizoma* Nakai (Mian Ma Guan Zhong)	Yang et al. (2013)
Respiratory syncytial virus	*Lonicera japonica* Thunb. Extracts	Inhibits viral absorption	*Lonicera japonica* Thunb. (Jin Yin Hua)	Zhang et al. (2014)
	*Ephedra Sinica* water extracts	Inhibits viral absorption and penetration	*Ephedra sinica* Stapf (Ma Huang)	Zhu and Li (2012)
	*Radix Glycyrrhizae* water extracts	Inhibits viral attachment and penetration	*Glycyrrhiza glabra* L. (Gan Cao)	Yeh et al. (2013)
SARS Coronavirus	Emodin	Targets spike glycoprotein thus inhibits receptor binding	*Rheum palmatum* L. (Da Huang)	Ho et al. (2007)

1.3 Inhibit viral replication and release				
Virus	Active component	Mechanisms	Herb	Ref
Bovine viral diarrhea virus, a surrogate in vitro model of hepatitis C virus	Novel artemisinin derivatives (AD)	AD1 and AD2 inhibit the release of Bovine viral diarrhea virus -RNA	*Artemisia annua* L. (Qing Hao)	Blazquez et al. (2013)
Coxsackie virus B3	Emodin	Unknown	*Rheum palmatum* L. (Da Huang)	Cai and Luo (2014)
	Artemisinin	Inhibits viral replication	*Artemisia annua* L. (Qing Hao)	Ma (2004)
	*Isatis tinctoria* L. polysaccharides extracts	Inhibits viral replication	*Isatis tinctoria* L. (Ban Lan Gen)	Zhang et al. (2009)
Coxsakievirus B5 and respiratory syncytial virus	Emodin	Inhibits Viral biological synthesis	*Rheum palmatum* L. (Da Huang)	Liu et al. (2015)
Dengue virus	*Lonicera japonica* Thunb. aqueous extracts	The microRNA let-7a targets viral non-structural protein1	*Lonicera japonica* Thunb. (Jin Yin Hua)	Lee et al. (2017)
Ebola virus	18β-glycyrrhetinic acid	Binds to nucleoprotein	*Glycyrrhiza glabra* L*.* (Gan Cao)	Fu et al. (2016)
Enterovirus 71	Glycyrrhizic acid	Inhibits viral replication	*Glycyrrhiza glabra* L*.* (Gan Cao)	Wang et al. (2013)
	*Rheum palmatum* L. extracts	Reduces viral replication	*Rheum palmatum* L. (Da Huang)	Lin et al. (2009)
	Norwogonin, oroxylin A, mosloflavone	Inhibits expression of viral capsid proteins	*Scutellaria baicalensis* Georgi (Huang Qin)	Choi et al. (2016)
	Baicalin	Interfers with 3D polymerase transcription and translation	*Scutellaria baicalensis* Georgi (Huang Qin)	Li et al. (2015)
	Honeysuckle-encoded microRNA2911	Targets viral envelope protein1 gene of Enterovirus 71	*Lonicera japonica* Thunb. (Jin Yin Hua)	Li et al. (2018)
	Emodin	Diminishes cell cycle arrest at S phase induced infection	*Rheum palmatum* L. (Da Huang)	Zhong et al. (2017)
Epstein-Barr Virus	Baicalein	Represses Epstein–Barr nuclear antigen1 Q-promoter activity	*Scutellaria baicalensis* Georgi (Huang Qin)	Zhang et al. (2018)
	5,7,2′-trihydroxy- and 5,7,2′,3′-tetrahydroxyflavone	Unknown	*Scutellaria baicalensis* Georgi (Huang Qin)	Konoshima et al. (1992)
	*Arctium lappa* L. extracts	Suppresses viral replication and decreases viral antigen expression, including capsid antigen and early antigen	*Arctium lappa* L. (Niu Bang Zi)	Chen and Huang (1994)
Hepatitis B virus	Novel artemisinin derivatives (AD)	AD1 and AD2 reduce the release of Hepatitis B virus -DNA	*Artemisia annua* L. (Qing Hao)	Blazquez et al. (2013)
Hepatitis C virus	Pheophytin	Inhibits Hepatitis C virus -nonstructural3 protease	*Lonicera japonica* Thunb. (Jin Yin Hua)	Wang et al. (2009a)
Herpes simplex virus	*Houttuynia cordata* Thunb. Extracts	Suppresses viral replication *via* inhibiting NF-κB activation	*Houttuynia cordata* Thunb. (Yu Xing Cao)	Hung et al. (2015)
Herpes simplex virus type1	*Isatis tinctoria* L. extracts	Inhibits viral replication	*Isatis tinctoria* L. (Ban Lan Gen)	Fang (2005)
	*Arctium lappa* L. hydroalcoholic extracts	Suppresses viral replication	*Arctium lappa* L. (Niu Bang Zi)	Dias et al. (2017)
	Chinonin/Asphonin	Inhibits viral replication	*Anemarrhena asphodeloides* Bunge (Zhi Mu)	Jiang and Xiang (2004)
Herpes simplex virus type2	Chinonin/Asphonin	Inhibits viral replication	*Anemarrhena asphodeloides* Bunge (Zhi Mu)	Jiang et al. (2005)
Human cytomegalovirus	Artemisinin-derived monomers artesunate (AS)	Inhibits viral replication as hypophosphorylation (activity) of the retinoblastoma protein (pRb)	*Artemisia annua* L. (Qing Hao)	Roy et al. (2015)
	Genistein	Blocks viral immediate-early protein functioning	*Scutellaria baicalensis* Georgi (Huang Qin)	Evers et al. (2005)
Human immunodeficiency virus type1	Artemisia afra	Unknown	*Artemisia annua* L. (Qing Hao)	Lubbe et al. (2012)
	Sennoside A	Inhibits viral replication by targeting viral reverse transcription process including inhibiting HIV-1 Reverse Transcriptase-associated DNA Polymerase and Ribonuclease H activities	*Rheum palmatum* L. (Da Huang)	Esposito et al. (2016)
	Baicalein	Binds to the hydrophobic region of the HIV-1 integrase catalytic core domain	*Scutellaria baicalensis* Georgi (Huang Qin)	Ahn et al. (2001)
	Baicalin	Inhibits HIV-1 reverse transcriptase activity	*Scutellaria baicalensis* Georgi (Huang Qin)	Kitamura et al. (1998)
	Containing *Scutellaria baicalensis* aqueous extracts	Inhibits human immunodeficiency virus type-1 protease	*Scutellaria baicalensis* Georgi (Huang Qin)	Lam et al. (2000)
Human rotavirus	*Rheum palmatum* L. extracts	Inhibits viral replication	*Rheum palmatum* L. (Da Huang)	He et al. (2013)
Influenza A Virus	*Isatis tinctoria* L. erucic acid	Reduces viral polymerase transcription activity	*Isatis tinctoria* L. (Ban Lan Gen)	Liang et al. (2020)
	Baicalein and biochanin A	Inhibits viral replication	*Scutellaria baicalensis* Georgi (Huang Qin)	Michaelis et al. (2014)
	Oroxylin A	Inhibits neuraminidase	*Scutellaria baicalensis* Georgi (Huang Qin)	Jin et al. (2018)
	Flavonoids-enriched extract from *Scutellaria baicalensis* root	Inhibits neuraminidase activities	*Scutellaria baicalensis* Georgi (Huang Qin)	Zhi et al. (2019)
	Baicalin	Inhibits RNA polymerase activity	*Scutellaria baicalensis* Georgi (Huang Qin)	Guo et al. (2016)
	Baicalin	Interacts with RNA binding domain of Non-structural protein1	*Scutellaria baicalensis* Georgi (Huang Qin)	Nayak et al. (2014)
	Glycyrrhizin	Inhibits influenza virus polymerase activity	*Glycyrrhiza glabra* L*.* (Gan Cao)	Moisy et al. (2012)
	Aloe-emodin	Inhibits viral replication through galectin-3 up-regulation	*Rheum palmatum* L. (Da Huang)	Li et al. (2014)
	Baicalin	Inhibits viral replication	*Scutellaria baicalensis* Georgi (Huang Qin)	Sithisarn et al. (2013)
	Baicalin	Inhibits neuraminidase activity	*Scutellaria baicalensis* Georgi (Huang Qin)	Sithisarn et al. (2013)
	*Isatis tinctoria* L. extract Clemastanin B (CB), epigoitrin, phenylpropanoids portion (PEP) and the mixture of phenylpropanoids, alkaloids and organic acid fractions	Inhibits viral replication	*Isatis tinctoria* L. (Ban Lan Gen)	Xiao et al. (2016)
	*Isatis tinctoria* L. extracts	Suppresses expression of influenza virus nucleoprotein	*Isatis tinctoria* L. (Ban Lan Gen)	Xu et al. (2010)
	*Pogostemon cablin* (Blanco) Benth extracts	Suppresses viral replication	*Pogostemon cablin* (Blanco) Benth. (Guang Huo Xiang)	Yang (2010)
	Fritillaria thunbergii	Unknown	*Fritillaria thunbergii* Miq. (Zhe Bei Mu)	Kim et al. (2020)
	Chlorogenic acid	Inhibits neuraminidase	*Lonicera japonica* Thunb. (Jin Yin Hua)	Ding et al. (2017)
	Honeysuckle (HS)-encoded atypical microRNA-MIR2911	Inhibits IAV-encoded PB2 and NS1 protein expression	*Lonicera japonica* Thunb. (Jin Yin Hua)	Zhou et al. (2015)
	Forsythoside A from *Forsythia suspensa* (Thunb.) Vahl fruit	Reduces influenza viral M1 protein	*Forsythia suspensa* (Thunb.) Vahl (Lian Qiao)	Law et al. (2017)
	Chalcones	Inhibits neuraminidase activity	*Glycyrrhiza glabra* L*.* (Gan Cao)	Dao et al. (2011)
	*Houttuynia cordata* Thunb. flavonoids extracts	Inhibits neuraminidase activity	*Houttuynia cordata* Thunb. (Yu Xing Cao)	Ling et al. (2020)
	*Isatis tinctoria* L. N-butanol extracts	Inhibits viral replication	*Isatis tinctoria* L. (Ban Lan Gen)	Liu et al. (2012)
Newcastle disease virus	Baicalin	Inhibits apoptosis of virus-infected cells and suppresses viral spread	*Scutellaria baicalensis* Georgi (Huang Qin)	Jia et al. (2016)
Polyphenolic extracts	*Pogostemon cablin* (Blanco) Benth polyphenolic extracts	Inhibits neuraminidase activity	*Pogostemon cablin* (Blanco) Benth. (Guang Huo Xiang)	Liu (2016)
Porcine epidemic diarrhea virus	*Pogostemon cablin* (Blanco) Benth polysaccharides extracts	Inhibits viral replication	*Pogostemon cablin* (Blanco) Benth. (Guang Huo Xiang)	Chen et al. (2020)
Porcine reproductive and respiratory syndrome virus	*Isatis tinctoria* L. polysaccharide extracts	Inhibits viral replication	*Isatis tinctoria* L. (Ban Lan Gen)	Wei et al. (2011)
	Flavaspidic acid AB from Dryopteris crassirhizoma	Inhibits viral replication	*Dryopteris crassirhizoma* Nakai (Mian Ma Guan Zhong)	Yang et al. (2013)
	*Isatis tinctoria* L. *polysaccharide* extracts	Inhibits viral replication	*Isatis tinctoria* L. (Ban Lan Gen)	Liu (2016)
	Artemisinin	Inhibits viral replication	*Artemisia annua* L. (Qing Hao)	Liu (2016)
Respiratory syncytial virus	Isatis root extract	Inhibits viral NS1 and L proteins	*Isatis tinctoria* L. (Ban Lan Gen)	Zhang (2017)
	(-)-(R)-nyasol (= 4,4'-(1Z,3R)-Penta-1,4-diene-1,3-diyldiphenol and broussonin A	Unknown	*Anemarrhena asphodeloides* Bunge (Zhi Mu)	Bae et al. (2007)
	*Lonicera japonica* Thunb. Extracts	Inhibits viral biosynthesis	*Lonicera japonica* Thunb. (Jin Yin Hua)	Li (2010)
SARS coronavirus	*Houttuynia cordata* Thunb. Extracts	Inhibits SARS-CoV 3C-like protease and RNA-dependent RNA polymerase	*Houttuynia cordata* Thunb. (Yu Xing Cao)	Lau et al. (2008)
	Rheum palmatum L. extracts	Inhibits SARS coronavirus 3C-like protease	*Rheum palmatum* L. (Da Huang)	Luo et al. (2009)

**TABLE 2:** Active anti-viral components from LHQWC and JHQGG regulating host immune responses and inflammation.

Virus	Active component	Mechanisms	Herb	References
Bovine viral diarrhea virus	Forsythoside A	Promotes peripheral blood mononuclear cell proliferation and T cell activation, TRAF2-dependent CD28-4-1BB signaling; induces IFN-γ	*Forsythia suspensa* (Thunb.) Vahl (Lian Qiao)	Li et al. (2011)
Coxsackie virus B3	Emodin	Reduces pro-inflammatory cytokines	*Rheum palmatum* L. (Da Huang)	Cai and Luo (2014)
	Emodin	Regulates IL-17/IL-23 axis	*Rheum palmatum* L. (Da Huang)	Jiang et al. (2014)
	Rhodiola	Unknown	*Rhodiola crenulata* (Hook.f. and Thomson) H.Ohba (Hong Jing Tian)	Liu et al. (2002)
Coxsakievirus B5 and respiratory syncytial virus	Emodin	Decreases IFN-α, enhance TNF-γ	*Rheum palmatum* L. (Da Huang)	Liu et al. (2015)
Hepatitis B virus	*Isatis tinctoria* L. polysaccharide extracts	Enhances IFN-α and antiviral proteins, including p-STAT-1, p-STAT-2, p-JAK1, p-TYK2, OAS1, and Mx, *via* activation of JAK/STAT signal pathway	*Isatis tinctoria* L. (Ban Lan Gen)	Wang et al. (2020b)
Hepatitis C virus	Artemisia annua polysaccharides	Promotes IFN-γ secretion	*Artemisia annua* L. (Qing Hao)	Bao et al. (2015)
Herpes simplex virus type1	Essential oil of Mentha suaveolens	Unknown	*Mentha canadensis* L. (Bohe)	Civitelli et al. (2014)
Influenza A Virus	*Isatis tinctoria* L.erucic acid	Reduces viral RNA-induced pro-inflammatory mediators through inactivation of NF-κB and p38 MAPK signaling pathway, Reduce CD8 (+) cytotoxic T lymphocyte recruitment	*Isatis tinctoria* L. (Ban Lan Gen)	Liang et al. (2020)
	Oroxylin A	Increases IFN-β and IFN-γ	*Scutellaria baicalensis* Georgi (Huang Qin)	Jin et al. (2018)
	Flavonoids-enriched extract from *Scutellaria baicalensis* root	Reduces TNF-α, IL-6 and MCP-1, increases IFN-γ and IL-10	*Scutellaria baicalensis* Georgi (Huang Qin)	Zhi et al. (2019)
	Baicalin	Modulates non-structural protein1-mediated cellular innate immune responses, IFN-induced antiviral signaling and a decrease in PI3K/Akt signaling	*Scutellaria baicalensis* Georgi (Huang Qin)	Nayak et al. (2014)
	Phillyrin	Decreases IL-6	*Forsythia suspensa* (Thunb.) Vahl (Lian Qiao)	Qu et al. (2016)
	Aloe-emodin	Restores NS1-inhibited STAT1-mediated antiviral responses	*Rheum palmatum* L. (Da Huang)	Li et al. (2014)
	Ephedra alkaloids: L-ephedrine and D-pseudo- ephedrine	Regulating TLRs and RIG-1 pathways	*Ephedra sinica* Stapf (Ma Huang)	Wei et al. (2019)
	*Radix Isatidis* extract	Promotes T, B lymphocytes	*Isatis tinctoria* L. (Ban Lan Gen)	Jin (2007)
	*Radix Isatidis* polysaccharides	Promotes IFN-γ secretion	*Isatis tinctoria* L. (Ban Lan Gen)	Zuo (2008)
	Salidroside	Reduces IL1-β, IL-6, TNF-α and CRP, increases the number of CD4 (+) T cells	*Rhodiola crenulata* (Hook.f. and Thomson) H.Ohba (Hong Jing Tian)	Lin (2020)
	Baicalin	Balances host inflammatory response to limit immunopathologic injury; downregulated the key factors of the RLRs signaling pathway	*Scutellaria baicalensis* Georgi (Huang Qin)	Pang et al. (2018)
	Baicalin	Inhibits TLR7/MyD88 signaling pathway	*Scutellaria baicalensis* Georgi (Huang Qin)	Wan et al. (2014)
	Biochanin A	Reduces AKT, ERK 1/2 and NF-kB	*Scutellaria baicalensis* Georgi (Huang Qin)	Sithisarn et al. (2013)
	Biochanin A	Inhibits IL-6, IL-8 and IP-10	*Scutellaria baicalensis* Georgi (Huang Qin)	Sithisarn et al. (2013)
	Baicalin	Inhibits IL-6 and IL-8	*Scutellaria baicalensis* Georgi (Huang Qin)	Sithisarn et al. (2013)
	*Radix Isatidis* polysaccharides	Suppresses pro-inflammatory IL-6 and chemokines (IP-10, MIG, and CCL-5), inhibits host TLR3 Signaling	*Isatis tinctoria* L. (Ban Lan Gen)	Li et al. (2017b)
	Wogonin	Reduces inflammatory factors	*Scutellaria baicalensis* Georgi (Huang Qin)	Wu (2011)
	Epigoitrin	Reduces mitochondria mitofusin-2, which elevated mitochondria antiviral signaling and subsequently increased IFN-β and interferon inducible transmembrane 3 (IFITM3)	*Isatis tinctoria* L. (Ban Lan Gen)	Luo et al. (2019)
	Rhein	Activates TLR4, Akt, p38, JNK MAPK, and NF-κB signal pathways	*Rheum palmatum* L. (Da Huang)	Wang et al. (2018)
	Baicalin	Reduces TNF-α,IL-1 and 5-HT; increases IFN-γ	*Scutellaria baicalensis* Georgi (Huang Qin)	Li (2019)
	*Isatis tinctoria* L.extracts	Regulates immune response by enhancing proliferation and function of T and B cells	*Isatis tinctoria* L. (Ban Lan Gen)	Jin (2007)
	Dryocrassin ABBA	Decreases bronchoalveolar lavage fluid pro-inflammatory cytokines, including IL-6, TNF-α, and IFN-γ, and increases anti-inflammatory cytokines, including IL-10 and MCP-1	*Dryopteris crassirhizoma* Nakai (Mian Ma Guan Zhong)	Ou et al. (2015)
	Baicalin	Imcreases IFN-γ production	*Scutellaria baicalensis* Georgi (Huang Qin)	Chu et al. (2015)
	*Lonicera Japonica* Thunb polysaccharide	Increases IFN-γ	*Lonicera japonica* Thunb. (Jin Yin Hua)	Jia (2018)
	*Lonicera Japonica* water decoction	Increases IFN-γ	*Lonicera japonica* Thunb. (Jin Yin Hua)	Zhu (2016)
	*Lonicerae Japonicae* Los and Forsythiae Fructus	Modulates MMP pathway and PRKCA pathway	*Lonicera japonica* Thunb. (Jin Yin Hua)	Li (2017)
	Forsythoside A	Reduces TLR7, MyD88 and NF-κB p65 protein; Inducing Th1/Th2 differentiats toward Th2, and the Th17/Treg cells differentiates toward Treg	*Forsythia suspensa* (Thunb.) Vahl (Lian Qiao)	Deng et al. (2016)
	Ethanol extracts of *Forsythia suspensa* Vahl. (Oleaceae), *Strobilanthes cusia* (Ness.) O. Kuntze (Acanthaceae), *Glycyrrhiza uralensis Fischer*. (Leguminosae)	Suppresses RANTES secretion	*Forsythia suspensa* (Thunb.) Vahl (Lian Qiao) *Isatis tinctoria* L. (Ban Lan Gen) *Glycyrrhiza glabra* L*.* (Gan Cao)	Ko et al. (2006)
	*Houttuynia cordata* Thunb. flavonoids extracts	Inhibits TLR signaling, increases IFN-β, decreases of TLR3/4/7 and NF-κB p65(p), MCP-1), IL-8, TNF-α and MDA	*Houttuynia cordata* Thunb. (Yu Xing Cao)	Ling et al. (2020)
Influenza A Virus and Influenza B Virus	Wogonin	Increases IFN	*Scutellaria baicalensis* Georgi (Huang Qin)	Seong et al. (2018)
Japanese encephalitis virus	Arctigenin	Anti-inflammatory	*Arctium lappa* L. (Niu Bang Zi)	Swarup et al. (2008)
Porcine reproductive and respiratory syndrome virus	Flavaspidic acid AB	Induces IFN-α, IFN-β, and IL1-β expression in porcine alveolar macrophages	*Dryopteris crassirhizoma* Nakai (Mian Ma Guan Zhong)	Yang et al. (2013)
Respiratory Syncytial Virus	Baicalin	Increases IFN-1, decreases IL-6, IL-12	*Scutellaria baicalensis* Georgi (Huang Qin)	Zhang (2018)
	Rhein	Inhibits NLRP3 inflammasome activation through NF-kB pathway	*Rheum palmatum* L. (Da Huang)	Shen et al. (2020)
	4(3H)-Quinazolone	Inhibits IFN-β secretion	*Isatis tinctoria* L. (Ban Lan Gen)	He et al. (2017)
	Total alkaloids, lignans and organic acids of *Radix Isatidis* extracts	Regulates IFNβ, synergistic effects through RIG-I and MDA5 signaling pathways	*Isatis tinctoria* L. (Ban Lan Gen)	Xu et al. (2019)
	Baicalin joint resveratrol	Increase serum TNF-α, IL-2, IFN-γ and SIgA in bronchoalveolar lavage fluid	*Scutellaria baicalensis* Georgi (Huang Qin)	Cheng et al. (2014)
	*Radix Glycyrrhizae* water extracts	Induces IFN-β secretion	*Glycyrrhiza glabra* L*.* (Gan Cao)	Yeh et al. (2013)
SARS coronavirus	*Houttuynia cordata* Thunb. Extract	Immunomodulatory effects: stimulating mouse splenic lymphocytes the proliferation and increasing the proportion of CD4 (+) and CD8 (+) T cells, increases secretion of IL-2 and IL-10 by mouse splenic lymphocytes	*Houttuynia cordata* Thunb. (Yu Xing Cao)	Lau et al. (2008)
Vesicular stomatitis virus	Extract from *Scutellaria baicalensis* containing baicalein and wogonin	Inhibits IFN-alpha and IFN- γ, and stimulates TNF-α and IL (IL-12, IL-10) production	*Scutellaria baicalensis* Georgi (Huang Qin)	Blach-Olszewska et al. (2008)
	Baicalin	Increases IFN-γ, reduces TNF-α and IL-10	*Scutellaria baicalensis* Georgi (Huang Qin)	Orzechowska et al. (2014)

IFN, Interferon; IL, Interleukin; MCP-1 Monocyte chemoattractant protein-1; MDA5, Melanoma differentiation-associated protein 5; MIG, Monokine induced by gamma interferon; MMP, Matrix metalloproteinases; MYD88, Myeloid differentiation factor 88; NLRP3, NLR Family Pyrin Domain Containing 3; PRKCA, Protein Kinase C Alpha; RANTES, Regulated upon activation, normal T cell expressed and presumably secreted; RIG-I, Retinoic acid-inducible gene I; STAT, Signal transducer and activator of transcription; TLR, Toll-like receptor; TNF, Tumor Necrosis Factor; TRAF2, TNF Receptor-associated Factor 2; 5-HT, 5-hydroxytryptamine.

**TABLE 3:** Active anti-viral components from LHQWC and JHQGG regulating host redox homeostasis and other molecular actions.

3.1 Regulate redox homeostasis				
Virus	Active component	Mechanisms	Herb	References
*Herpes simplex virus type1*	Piperitenone oxide	Interferes with redox-sensitive cellular pathways for viral replication	*Mentha canadensis* L. (Bohe)	Civitelli et al. (2014)
*Japanese encephalitis virus*	Arctigenin	Promotes antioxidative effects	*Arctium lappa* L. (Niu Bang Zi)	Swarup et al. (2008)
*Influenza A Virus*	Oroxylin A	Activates the nuclear factor erythroid 2–related factor 2 (Nrf2) transcription to increase antioxidant activities	*Scutellaria baicalensis* Georgi (Huang Qin)	Ji et al. (2015)
	Rhein	Reduces antioxidative stress	*Rheum palmatum* L. (Da Huang)	Wang et al. (2018)
*Coxsackie virus B3*	Emodin	Up-regulates anti-oxidant enzymes	*Rheum palmatum* L. (Da Huang)	Cai and Luo (2014)
	*Isatis tinctoria* L. Salidroside	Increases myocardial SOD activity and decreases MDA	*Isatis tinctoria* L. (Ban Lan Gen)	Wang et al. (2009b)
	Honeysuckle	Inhibits oxidative stress	*Lonicera japonica* Thunb. (Jin Yin Hua)	Lou (2017)
*Porcine epidemic diarrhea virus*	*Pogostemon cablin* (Blanco) Benth polysaccharides extracts	Increases SOD and GSH-Px activity and decreases MDA	*Pogostemon cablin* (Blanco) Benth. (Guang Huo Xiang)	Wang (2010)
*Hepatitis C virus*	A glycyrrhizin-containing preparation	Protects mitochondria against oxidative stress	*Glycyrrhiza glabra* L*.* (Gan Cao)	Korenaga et al. (2011)

3.2 Other molecular actions				
Virus	Active component	Mechanisms	Herb	References
Enterovirus 71	Baicalin	Inhibits virus-induced apoptosis through regulating the Fas/FasL signaling pathways	*Scutellaria baicalensis* Georgi (Huang Qin)	Li et al. (2015)
Influenza A Virus	*Houttuynia cordata* Thunb. polysaccharide extracts	Acts on intestine and microbiota	*Houttuynia cordata* Thunb. (Yu Xing Cao)	Chen et al. (2019)
	*Houttuynia cordata* Thunb	Protects intestinal barrier and regulates mucosal immunity, which may be related to the regulation of gut-lung axis	*Houttuynia cordata* Thunb. (Yu Xing Cao)	Zhu et al. (2018)
	Baicalin	Reduces endothelin (ET-1) and ET-1 receptor	*Scutellaria baicalensis* Georgi (Huang Qin)	Wan (2015)
	*Houttuynia cordata* Thunb.polysaccharides	Regulates the balance of Th17/Treg cells in gut-lung axis	*Houttuynia cordata* Thunb. (Yu Xing Cao)	Shi et al. (2020)
Influenza A Virus and influenza B Virus	Wogonin	Suppresses AMPK phosphorylation	*Scutellaria baicalensis* Georgi (Huang Qin)	Seong et al. (2018)
Human cytomegalovirus	Baicalin	Regulates vasoactive intestinal peptide	*Scutellaria baicalensis* Georgi (Huang Qin)	Qiao et al. (2013)
	Artemisinin	Modulates cell cycle through CDKs and hypophosphorylation (activity) of the retinoblastoma protein (pRb)	*Artemisia annua* L. (Qing Hao)	Roy et al. (2015)
Herpes simplex virus type 1	Triterpene glycyrrhizic acid	Induces autophagy activator Beclin 1 to establish a resistance state to viral replication	*Glycyrrhiza glabra* L*.* (Gan Cao)	Laconi et al. (2014)

GSH-Px, Glutathione peroxidase; MDA, Malondialdehyde; SOD, Superoxide dismutase.

AMPK, AMP-activated protein kinase; CDKs, Cyclin-dependent kinases; Th17/Treg, T helper 17 (Th17)/regulatory T cells (Tregs).

**TABLE 4:** Detailed information of TCM features and pharmacological functions of single medicinal herbs from LHQWC and JHQGG.

4.1 Specific medicinal herbs of LHQWC							
Components of medicinal herbs	TCM properties	Key characteristics	Active component	Virus	Pharmacological functions	References
*Rheum palmatum* L. (Da Huang)	Bitter	Purges clumped heat in the intestines	Emodin	Coxsackie virus B3	Decreases overall mortality of virus-induced murine viral myocarditis model and potentially could act through inhibiting viral replication, reducing pro-inflammatory cytokines and up-regulation of anti-oxidant enzymes	Cai and Luo (2014)
	Cold	Removes blood stasis			Reduces mice mortality rate and ameliorates myocardial damage by regulating the IL-17/IL-23 axis	Jiang et al. (2014)
		Stops bleeding in its charred form		Coxsackie virus B5	Inhibits activities against coxsackie virus B5	Liu et al. (2015)
				Enterovirus 71	Inhibits viral replication and diminishes cell cycle arrest at S phase induced by EV71 infection in MRC5 cells	Zhong et al. (2017)
			Aloe-emodin	Influenza A Virus	Inhibits viral replication through galectin-3 up-regulation	Li et al. (2014)
			Rhein	Respiratory syncytial virus	Suppresses lung inflammatory injury by reducing the release of pro-inflammatory cytokines, including IL-1β, IL-6, TNF-α, IL-18, and IL-33, in the serum and lung tissues of RSV-induced BALB/c mice through inhibiting NLRP3 inflammasome activation *via* NF-κB pathway	Shen et al. (2020)
				Influenza A virus	Inhibits viral absorption	Wang et al. (2018)
			Sennoside A	Human immunodeficiency virus type1	Inhibits the HIV-1 replication by targeting the HIV-1 reverse transcription process including inhibiting HIV-1 Reverse Transcriptase-associated DNA Polymerase and Ribonuclease H activities	Esposito et al. (2016)
			Extracts	SARS coronavirus	Inhibits SARS coronavirus 3C-like protease	Luo et al. (2009)
				Rotavirus	Inhibits viral entry and replication in MA-104 cells	He et al. (2013)
*Houttuynia cordata* Thunb*.*(Yu Xing Cao)	Acrid	Disperses heat	Houttuynoid A	Herpes simplex virus type 1	Exhibits strong antiviral activity including inhibiting viral replication, inactivating viral infectivity by blocking viral membrane fusion and preventing lesion formation in HSV-1 infection mouse model. It also exhibits antiviral activities against other alpha herpes viruses, such as HSV-2 and varicella zoster virus	Li et al. (2017a)
	Cool	Resolves toxicity	Polysaccharides extracts	Influenza A virus	Oral administration could ameliorate lung injury in virus-infected mice via directly regulating the balance of Th17/Treg cells in gut-lung axis	Shi et al. (2020)
		Reduces swelling			Acts on intestine and microbiota	Chen et al. (2019)
			Flavonoids extracts	Influenza A virus	Significantly inhibit viral proliferation and suppress neuraminidase activity and TLR3, TLR4, and TLR7 agonist-stimulated cytokine secretion, NF-κB p65 phosphorylation, and nuclear translocation *in vitro*	Ling et al. (2020)
			Extracts	Influenza A virus	Protects intestinal barrier and regulates mucosal immunity, which may be related to the regulation of gut-lung axis	Zhu et al. (2018)
				Enterovirus 71	Reduces plaque formation and neutralizes virus-induced cytopathic effects in Vero cells and could affect apoptotic processes in virus-infected Vero cells by inhibiting viral replication	Lin et al. (2009)
				SARS coronavirus	Exerts anti-viral effects, including inhibitory effects on SARS-CoV 3C-like protease and RNA-dependent RNA polymerase. Exhibits immunomodulatory effects, including stimulating the proliferation of mouse splenic lymphocytes and increasing the proportion of CD4 (+) and CD8 (+) T cells and the secretion of IL-2 and IL-10 by mouse splenic lymphocytes	Lau et al. (2008)
				Herpes simplex virus	Inhibits the infection of HSV-1, HSV-2, and acyclovir-resistant HSV-1 *via* blocking viral binding and penetration. Suppresses viral replication via inhibiting NF-κB activation	Hung et al. (2015)
*Isatis tinctoria* L*.* (Ban Lan Gen)	Bitter	Drains heat	Erucic acid	Influenza A virus	Suppresses viral replication by reducing viral polymerase transcription activity and inhibits RNA-induced pro-inflammatory mediators through inactivation of NF-κB and p38 MAPK signaling pathway. Inhibits alveolar epithelial A549 cells apoptosis. Decreases lung viral load and viral antigens expression, and reduces CD8 (+) cytotoxic T lymphocyte recruitment, which results in decreasing lung injury and mortality of virus-infected mice	Liang et al. (2020)
	Cold	Resolves fire toxicity	Epigoitrin	Influenza A virus	Reduces mitochondria mitofusin-2, which elevated mitochondria antiviral signaling and subsequently increased IFN-β and interferon inducible transmembrane 3	Luo et al. (2019)
		Cools the blood	4(3H)-Quinazolone	Respiratory Syncytial Virus	Inhibits IFN-β secretion	He et al. (2017)
		Benefits the throat	Clemastanin B, epigoitrin, phenylpropanoids portion and the mixture of phenylpropanoids, alkaloids and organic acid fractions	Influenza A virus	Inhibits viral replication, entry and improves the viability of infected MDCK cells	Xiao et al. (2016)
			Polysaccharide extracts	Influenza A virus	Inhibits virus replication and reduces the expression of pro-inflammatory cytokines (IL-6) and chemokines (IP-10, MIG, and CCL-5) by inhibiting TLR-3 signaling pathway activation	Li et al. (2017b)
				Hepatitis B virus	Reduce extracellular and intracellular level of HBsAg, HBeAg and HBV DNA and enhance the production of IFN-α and antiviral proteins, including p-STAT-1, p-STAT-2, p-JAK1, p-TYK2, OAS1, and Mx, *via* activation of JAK/STAT signal pathway	Wang et al. (2020c)
				Influenza A virus	Promotes IFN-γ secretion	Zuo (2008)
			N-butanol extract	Influenza A virus	The metabolites of extract inhibit the neuraminidase activities	Liu et al. (2012)
			Extracts	Respiratory syncytial virus	Relieves virus-induced mouse lung lesions and regulates the expression levels of IFN-β and inflammatory cytokines between antiviral and proinflammatory effects via the RIG-I and MDA5 signaling pathways	Xu et al. (2019)
					Inhibits viral NS1 and L proteins	Zhang (2017)
				Influenza A virus	Pretreatment with extract inhibits virus-cell adhesion	Chen et al. (2006)
					Suppresses the expression of influenza virus nucleoprotein	Xu et al. (2010)
					Promotes T, B lymphocytes	Jin (2007)
					Inhibits viral entry and impedes viral replication	Fang (2005)
					Alleviate the symptoms of virus-infected mice and regulates the immune response by enhancing proliferation and function of T and B cells	Jin (2007)
*Rhodiola crenulata* (Hook.f. and Thomson) H.Ohba (Hong Jing Tian)	Sweet	Raises qi	Salidroside	Influenza A virus	Relieves lung inflammation in infected mice and reduce the level of inflammatory factors, including IL-1β, IL-6, TNF-α, and C-reactive protein in both serum and lung tissue. Increases the number of CD4 (+) T cells	Lin (2020)
	Bitter	Invigorates the blood	Salidroside	Coxsackievirus B3	Decreases LDH release of infected cardiomyocytes and increase myocardial SOD activity and decreases MDA concentration of CVB3-induced viral myocarditis mice	Wang et al. (2009a)
	Neutral	Alleviate cough	Rhodiola	Coxsackievirus B3	Decreases LDH release of CVB3-infected viral myocarditis mice	Liu et al. (2002)
			Polysaccharides extract	Coxsackievirus B3	Inhibits viral replication and protect cardiomyocytes against virus-induced cell apoptosis	Zhang et al. (2009)
*Pogostemon cablin* (Blanco) Benth*.* (Guang Huo Xiang)	Acrid	Transform turbidity with aroma	Patchouli alcohol	Influenza A virus	Inhibits viral infection at the earliest stages of the viral life cycle, including virus attachment and entry	Wei et al. (2013)
	Slightly	Check retching		Coxsackievirus B3		
	Warm	Resolve summerheat		Adenovirus		
			Polyphenolic extracts	Influenza A virus	Inhibits neuraminidase activity	Liu (2016)

4.2 Specific medicinal herbs of JHQGG							
Components of medicinal herbs	TCM properties	Key characteristics	Active component	Virus	Pharmacological functions		Referemces
*Dryopteris crassirhizoma* Nakai (Mian Ma Guan Zhong)	Bitter	Clears internal heat toxin	Dryocrassin ABBA	Influenza A virus	Decreases lung index and virus loads and improves survival rate of H5N1-infected mice. Decreases levels of bronchoalveolar lavage fluid pro-inflammatory cytokines, including IL-6, TNF-α, and IFN-γ, and increases level of anti-inflammatory cytokines, including IL-10 and MCP-1	Ou et al. (2015)	
		Stops bleeding					
	Cold	Kills parasites	Extracts	Influenza A virus	Prevents viral infection and suppresses viral replication	Yang (2010)	
*Arctium lappa* L. (Niu Bang Zi)	Acrid	Disperses heat in the exterior and clears internal heat toxin	Arctiin	Influenza A virus	Arctigenin could inhibit viral replication and suppress the release of progeny viruses from the host cells. The combination of arctiin and oseltamivir could decrease the virus yields in both bronchoalveolar lavage fluids and lungs than the H1N1-infected mice treated with arctiin or oseltamivir alone	Hayashi et al. (2010)	
	Bitter	Benefits the throat	Arctigenin				
	Cold		Arctigenin	Japanese encephalitis virus	Anti-inflammatory	Swarup et al. (2008)	
			Hydroalcoholic extracts containing arctiin and arctiin	Herpes simplex virus type 1	Suppress viral replication	Dias et al. (2017)	
			Extracts	Epstein–Barr virus	Suppresses viral replication and decreases viral antigen expression, including capsid antigen and early antigen	Chen and Huang (1994)	
*Anemarrhena asphodeloides* Bunge (Zhi Mu)	Bitter	Clears fire and nourishes the Yin of the Lungs, Stomach, and Kidneys	Chinonin	Herpes simplex virus type 2	Suppresses viral entry and replication	Jiang et al. (2005)	
	Sweet			Herpes simplex virus type 1	Inhibits viral replication	Jiang and Xiang (2004)	
	Cold		(—)-(R)-nyasol	Respiratory syncytial virus	Suppresses viral replication more effective than ribavirin	Bae et al. (2007)	
			(—)-(R)-4’-O-methylnyasol				
			Broussonin A				
*Artemisia annua* L*.* (Qing Hao)	Bitter	Clears all types of yin level heat without injuring the qi, blood, or Yin	Artemisinin	Coxsackievirus B3	Inhibits viral replication	Ma (2004)	
	Cold			Cytomegalovirus	Induces early G1 arrest and prevent the progression of cell cycle toward the G1/S checkpoint through reducing the expression of cyclin-dependent kinases 2, 4, and 6 in CMV-infected cells	Roy et al. (2015)	
			Artemisia afra	Human immunodeficiency virus type1	Inhibits viral replication and release	Lubbe et al. (2012)	
			Polysaccharides extracts	Hepatitis C virus	Acts as an adjuvant in boosting the immune response and promote IFN-γ secretion	Bao et al. (2015)	
*Scutellaria baicalensis* Georgi (Huang Qin)	Bitter	Cools heat	Baicalein	Influenza A virus	Suppresses H5N1 replication with antioxidant N-acetyl-l-cysteine combination	Michaelis et al. (2014)	
	Cold	Dries dampness		Cytomegalovirus	Inhibits viral replication, reduces the levels of virus immediate-early proteins and blocks the nuclear translocation	Evers et al. (2005)	
		Stops bleeding			Inhibits viral replication and the expression of vasoactive intestinal peptide in virus-infected human trophoblast cell line	Qiao et al. (2013)	
		Quiets the fetus in pregnancy		Epstein-Barr Virus	Represses Epstein–Barr nuclear antigen1 and Q-promoter activity	Zhang et al. (2018)	
				Human immunodeficiency virus type 1	Binds to the hydrophobic region of the HIV-1 integrase catalytic core domain	Ahn et al. (2001)	
			Baicalin	Influenza A virus	Protects mice from infection by H1N1 associated with increasing IFN-γ production	Chu et al. (2015)	
					Inhibits virus replication and downregulates the key factors of the RLRs signaling pathway, including RIG-I and NF-κB p65 protein, in H1N1 infected mice	Pang et al. (2018)	
					Inhibits RNA polymerase activity	Guo et al. (2016)	
					Interacts with RNA binding domain of Non-structural protein1	Nayak et al. (2014)	
					Inhibits viral replication and neuraminidase activity	Sithisarn et al. (2013)	
					Inhibits TLR7/MyD88 signaling pathway	Wan et al. (2014)	
					Reduces TNF-α,IL-1 and 5-HT; increases IFN-γ	Li (2019)	
					Reduces endothelin (ET-1) and ET-1 receptor	Wan (2015)	
				Chikungunya Virus	Exhibits virucidal activity	Oo et al. (2018)	
				Coxsackie virus B3	Inhibits viral entry by reducing cellular lipid synthesis	Wang et al. (2020a)	
				Enterovirus 71	Inhibits viral replication and release by interfering with 3D polymerase transcription and translation	Li et al. (2015)	
				Human immunodeficiency virus type 1	Inhibits HIV-1 reverse transcriptase activity	Kitamura et al. (1998)	
				Respiratory Syncytial Virus	Increases IFN-1, decreases IL-6, IL-12	Zhang (2018)	
				Vesicular stomatitis virus	Increases IFN-γ, reduces TNF-α and IL-10	Orzechowska et al. (2014)	
			Baicalin joint resveratrol	Respiratory Syncytial Virus	Increases serum TNF-α, IL-2, IFN-γ and SIgA in bronchoalveolar lavage fluid	Cheng et al. (2014)	
			Wogonin	Influenza A virus	Suppresses both influenza A and B virus replication in MDCK and A549 cells	Seong et al. (2018)	
					Reduces inflammatory factors	Wu (2011)	
			5,7,4′-trihydroxy-8-methoxyflavone	Influenza A virus	Inhibits fusion of virus with endosome/lysosome membrane	Nagai et al. (1995a); Nagai et al. (1995b)	
			5,7,2′-trihydroxy- and 5,7,2′,3′-tetrahydroxyflavone	Epstein-Barr Virus	Inhibits viral replication and release	Konoshima et al. (1992)	
			Oroxylin A	Influenza A Virus	Inhibits neuraminidase	Jin et al. (2018)	
					Activates the nuclear factor erythroid 2–related factor 2 transcription to increase antioxidant activities	Ji et al. (2015)	
			Norwogonin, Oroxylin A, mosloflavone	Enterovirus 71	Inhibits expression of viral capsid proteins	Choi et al. (2016)	
			Artemisinin derivatives	Hepatitis B virus	Reduces viral release	Blazquez et al. (2013)	
			Extract containing baicalein and wogonin	Vesicular stomatitis virus	Inhibits IFN-α and IFN-γ, and stimulates TNF-α and IL (IL-12, IL-10) production	Blach-Olszewska et al. (2008)	
			Flavonoids-enriched extracts	Influenza A virus	Exhibits antiviral activity, including inhibiting viral replication in H1N1-infected MDCK cells, decreasing lung virus titers, reducing hemagglutinin titers and inhibiting neuraminidase activities in lungs of H1N1-infected mice	Zhi et al. (2019)	
			Aqueous extracts	Human immunodeficiency virus type 1	Inhibits HIV type-1 protease activities	Lam et al. (2000)	
*Fritillaria thunbergii* Miq*.* (Zhe Bei Mu)	Bitter	Cools heat	Extracts	Influenza A virus	Inhibits virus replication in embryonated eggs and reduces H1N1-infected mice mortality rate	Kim et al. (2020)	
	Cold	Transforms phlegm-heat					
		Releases constraint					
		Dissipates nodules, especially in the neck and breast					

4.3 Common medicinal herbs in both prescriptions						
Components of medicinal herbs	TCM properties	Key characteristics	Active component	Virus	Pharmacological functions	References
*Lonicera japonica* Thunb*.* (Jin Yin Hua)	Sweet	Disperses heat	Chlorogenic acid	Influenza A virus	Suppresses the nucleocapsid protein expression and the release of progeny viruses by inhibiting neuraminidase activity	Ding et al. (2017)
	Cold	Resolves toxicity	Pheophytin	Hepatitis C virus	Inhibits HCV viral proteins and RNA and exhibits synergistic anti-HCV activity with IFNα-2a	Wang et al. (2009b)
		Cools the blood	Honeysuckle-encoded atypical microRNA2911	Enterovirus 71	Inhibits EV71 replication by targeting the VP1 gene	Li et al. (2018)
		Stops bleeding		Influenza A virus	Inhibits H1N1, H5N1 and H7N9 viral replication and inhibits H1N1-encoded PB2 and NS1 protein expression. Reduces mouse mortality caused by H5N1 infection	Zhou et al. (2015)
			Polysaccharides extracts	Influenza A virus	Increases serum IFN-γ expression	Zhu (2016)
			Extracts	Respiratory Syncytial Virus	Inhibits virus attachment and replication in Hela cells	Li (2010)
				Dengue virus	Inhibits viral replication and release *via* the microRNA let-7a targeting viral non-structural protein 1	Lee et al. (2017)
				Coxsackie virus B3	Increases serum SOD activity and decreases MDA concentration of CVB3-induced viral myocarditis mice	Lou (2017)
*Ephedra sinica* Stapf (Ma Huang)	Acrid	Induces sweating	(+)-catechin	Influenza A virus	Suppresses viral replication by inhibiting acidification of endosomes and lysosomes	Mantani et al. (2001)
	Slightly bitter	Calms wheezing	L-methylephedrin, L-ephedrine, D-pseudo- ephedrine	Influenza A virus	Increases IFN-β and decreases TNF-α level by regulating TLRs and RIG-1 pathways	Wei et al. (2019)
	Warm	Promotes urination	Water Extract	Respiratory syncytial virus	Inhibits viral absorption and penetration	Zhu and Li (2012)
*Forsythia suspensa* (Thunb.) Vahl (Lian Qiao)	Bitter	Cools and vents heat, particularly in the Heart and upper burner	Forsythoside A	Influenza A virus	Inhibits virus spread by reducing influenza viral M1 protein	Law et al. (2017)
	Slightly acrid	Resolves toxicity			Reduces TLR7, MyD88 and NF-κB p65 protein	Deng et al. (2016)
	Slightly cold	Disperses clumps	Phillyrin	Influenza A virus	Decreases IL-6 levels, and reduces the expression of hemagglutinin in mice infected with influenza A virus	Qu et al. (2016)
*Mentha canadensis* L*.* (Bo He)	Acrid	Facilitates the dispersal of upper burner wind-heat	Essential oil extract, piperitenone oxide	Herpes simplex virus type 1	Inhibits viral replication	Civitelli et al. (2014)
	Aromatic	Cools and clears the eyes and head				
	Cooling	Soothers the throat				
		Facilitates the flow of Liver qi and expels turbid filth				
*Glycyrrhiza glabra* L*.* (Gan Cao)	Sweet	Tonifies the Spleen qi	Glycyrrhizin	Influenza A virus	Reduces endocytosis activity and virus uptake	Wolkerstorfer et al. (2009)
	Neutral	Moistens the Lungs			Inhibits influenza virus polymerase activity	Moisy et al. (2012)
		Moderates urgency and toxicity	Glycyrrhizic acid	Enterovirus 71	Inhibits viral replication	Wang et al. (2013)
		Drains fire	Chalcones	Influenza A virus	Inhibits neuraminidase activity	Dao et al. (2011)
			Triterpene glycyrrhizic acid	Herpes simplex virus type 1	Induces autophagy activator Beclin 1 to establish a resistance state to viral replication	Laconi et al. (2014)
			18β-glycyrrhetinic acid	Ebola virus	Binds to nucleoprotein	Fu et al. (2016)
			A glycyrrhizin-containing preparation	Hepatitis C virus	Protects mitochondria against oxidative stress	Korenaga et al. (2011)
			Water extracts	Respiratory syncytial virus	Induces IFN-β secretion	Yeh et al. (2013)
			Ethanol extracts	Influenza A virus	Suppresses RANTES secretion	Ko et al. (2006)

In terms of COVID-19, the ACE-2 has been identified as the most important receptor for SARS-CoV-2 viral entry, which constitutes the initial step of infection (Walls et al., 2020). Through informatic analysis, the *Rheum palmatum* L (Da Huang) in LHQWC was found to be able to suppress viral infection by directly blocking interactions between the spike protein and ACE2. In addition, in the SARS-CoV, MERS-CoV and other coronaviruses, the 3CL (3C-like) protease is one of the crucial enzymes that mediates viral replication and has been recognized as a potential therapeutic target (Pillaiyar et al., 2016; Galasiti Kankanamalage et al., 2018). These predictive evaluations showed that *Scutellaria baicalensis* Georgi (Huang Qin)*, Anemarrhena asphodeloides* Bunge (Zhi Mu) and *Arctium lappa* L (Niu Bang Zi) in JHQGG, as well as *Rheum palmatum* L (Da Huang) and *Houttuynia cordata* Thunb (Yu Xing Cao) in LHQWC can inhibit viral transcription and replication, especially that the *Rheum palmatum* L (Da Huang) in LHQWC was shown as a potential inhibitor of 3CL protease, suggesting underlying mechanisms of both LHQWC and JHQGG in the treatment of COVID-19.

Since LHQWC and JHQGG are both commonly used for the treatment of influenza in China, we additionally analyzed their possible roles in the inhibition of influenza viral invasion. Hemagglutinin (HA) on the surface of influenza virus is a tri-polymer, which promotes virus binding and entering into host cells. In contrast to HA, the neuraminidase (NA) of influenza viruses involves detachment and release of mature viruses from host cells (Gamblin and Skehel, 2010; Gaymard et al., 2016). Components of *Scutellaria baicalensis* Georgi (Huang Qin) of JHQGG have been shown to inhibit the whole life cycle of influenza viruses, such as inhibiting HA and NA, and suppressing replicons. Meanwhile, *Isatis tinctoria* L (Ban Lan Gen) and *Rheum palmatum* L (Da Huang) of LHQWC have also been reported to reduce the internalization and replication of influenza viruses. The shared herbs, such as *Ephedra sinica* Stapf (Ma Huang), *Lonicera japonica* Thunb (Jin Yin Hua), *Forsythia suspensa* (Thunb.) Vahl (Lian Qiao) and *Glycyrrhiza glabra* L (Gan Cao) in both LHQWC and JHQGG were experimentally proved as inhibitors of influenza virus life cycle (Table 1; Table 1).

## Discussion

In clinical practices of TCM, medicinal herbs are generally applied in the form of decoctions, which contain mixtures of a variety of herbs with different pharmacological functions. Instead of directly inactivating pathogens, therapeutic effects of TCM decoctions are achieved mainly through balancing host anti-viral responses and pathogenic factors. During COVID-19 epidemics, synergistic therapy of LHQWC with clinically approved reproposing antivirals, such as oseltamivir, umifenovir, ribavirin, lopinavir, peramivir, penciclovir or ganciclovir, has shown its advantages in improving associated symptoms and reducing the course of hospitalization and disease progression in several reported trials (Liu M. et al., 2020; Yu, 2020a; Yu, 2020b; Cheng, 2020; Hu et al., 2020; Li et al., 2020; Lv and Wang, 2020; Xiao et al., 2020; Chen, 2021; Liu et al., 2021). Similarly, combined anti-viral treatment with JHQGG in mild or moderate COVID-19 was beneficial in relieving clinical symptoms and reducing risks of severe COVID-19 (Liu Z. et al., 2020; Duan, 2020; Duan, 2020). These studies provide clinical evidence that combined treatment with either LHQWC or JHQGG is superior to conventional monotherapy of antivirals.

The primary conclusion of our study that both LHQWC and JHQGG are efficient for a large range of viral diseases has supported that TCM formulae can be potentially an alternative therapy for emerging viral diseases, especially when specific drugs and vaccines have not been fully developed and applied. However, when it comes to appropriate or precisive clinical applications of LHQWC and JHQGG, differences of their associated pharmacological actions turn out to be an essential point to be addressed. When comparing the anti-viral targets of LHQWC and JHQGG, both CPMs have been documented effective in interfering with viral components, with *Isatis tinctoria* L (Ban Lan Gen) and *Rheum palmatum* L (Da Huang) in LHQWC being the predominate viral inhibitors, followed by *Lonicera japonica* Thunb (Jin Yin Hua) and *Houttuynia cordata* Thunb (Yu Xing Cao). While in JHQGG, the *Scutellaria baicalensis* Georgi (Huang Qin) and subsequently *Lonicera japonica* Thunb (Jin Yin Hua) are the most important virucidal herbs. Typically, *Scutellaria baicalensis* Georgi (Huang Qin) of JHQGG have been highly nominated among all analyzed herbs contributing to suppression of the whole viral life cycle. Intriguingly, a direct virucidal activity was observed mostly in components from *Scutellaria baicalensis* Georgi (Huang Qin) and *Anemarrhena asphodeloides* Bunge (Zhi Mu) of JHQGG, though shared herbs, *Lonicera japonica* Thunb (Jin Yin Hua) and *Glycyrrhiza glabra* L (Gan Cao) were also involved. This set of data indicate that from the angle of viral life cycle, JHQGG may overweight LHQWC due to *Scutellaria baicalensis* Georgi (Huang Qin), and will be appropriate for patients with high fever, sore throat and cough. On the other hand, owning to existence of *Rhodiola crenu*lata (Hook.f. and Thomson) H. Ohba (Hong Jing Tian), LHQWC may have more essential roles in the balancing of host immunity, suggesting that LHQWC could be more suitable for patients with non-efficient anti-viral immune responses.

There are some possible limitations in this study. Firstly, based on five databases, we finally included relatively more articles associated with LHQWC compared with those of JHQGG; therefore, bias could be unintendedly introduced to conclusions supporting superiority of LHQWC. Secondly, a certain number of included studies focus on *Scutellaria baicalensis* Georgi (Huang Qin), *Isatis tinctoria* L (Ban Lan Gen) and *Rheum palmatum* L (Da Huang); therefore, this may lead to biases that only these herbs are important as antivirals. Thirdly, the quality of articles included in this study is variable, and the judgment for potential pharmacological actions may to some degree rely on the knowledge of authors.

COVID-19 initiates with mild or moderate symptoms in most cases, and the strategy to reduce risks in evolving into severe or critical COVID-19 is highly desired. Through literature mining, we provide general evidence that both LHQWC and JHQGG are effective for mild to moderate COVID-19 patients and potentially being able to prevent the progress of COVID-19 into severe or critical conditions. As discussed above, TCM therapy fits well with the principle of HDT, and anti-viral TCM formulae generally show a broad spectrum of anti-viral properties through balancing between viral activities and host immune reactions. This has gained TCM a key advantage over target-specific anti-viral medications. Since LHQWC and JHQGG are both CPMs with clear safety information, it is imperative that application of LHQWC and JHQGG can be contextualized to worldwide combat against the emerging or re-emerging of human pandemics.

## Data Availability

The original contributions presented in the study are included in the article, further inquiries can be directed to the corresponding authors.
